# Octopamine signaling via OctαR is essential for a well-orchestrated climbing performance of adult Drosophila melanogaster

**DOI:** 10.1038/s41598-022-18203-x

**Published:** 2022-08-18

**Authors:** Samar Ezzat El-Kholy, Basma Afifi, Iman El-Husseiny, Amal Seif

**Affiliations:** Zoology Department, Faculty of Science, Tanta University, Tanta, 31527 Egypt

**Keywords:** Experimental organisms, Developmental biology, Physiology

## Abstract

The biogenic amine octopamine (OA) orchestrates many behavioural processes in insects. OA mediates its function by binding to OA receptors belonging to the G protein-coupled receptors superfamily. Despite the potential relevance of OA, our knowledge about the role of each octopaminergic receptor and how signalling through these receptors controls locomotion still limited. In this study, RNA interference (RNAi) was used to knockdown each OA receptor type in almost all *Drosophila* melanogaster tissues using a tubP-GAL4 driver to investigate the loss of which receptor affects the climbing ability of adult flies. The results demonstrated that although all octopaminergic receptors are involved in normal negative geotaxis but Oct*α*R-deficient flies had impaired climbing ability more than those deficient in other OA receptors. Mutation in OA receptors coding genes develop weak climbing behaviour. Directing knockdown of oct*α*R either in muscular system or nervous system or when more specifically restricted to motor and gravity sensing neurons result in similar impaired climbing phenotype, indicating that within *Drosophila* legs, OA through Oct*α*R orchestrated the nervous system control and muscular tissue responses. Oct*α*R-deficient adult males showed morphometric changes in the length and width of leg parts. Leg parts morphometric changes were also observed in *Drosophila* mutant in *OctαR*. Transmission electron microscopy revealed that the leg muscles *OctαR*-deficient flies have severe ultrastructural changes compared to those of control flies indicating the role played by OctαR signalling in normal muscular system development. The severe impairment in the climbing performance of *OctαR*-deficient flies correlates well with the completely distorted leg muscle ultrastructure in these flies. Taken together, we could conclude that OA via *OctαR* plays an important multifactorial role in controlling locomotor activity of *Drosophila*.

## Introduction

Locomotor activity is an integrative characteristic of the functional state of the nervous system as it is implicated directly or indirectly in most types of insect behaviours such as foraging and mating. Distinct brain structures like the mushroom bodies or the central complex have been shown to be required for the control of locomotor activity^1,2^. In adult *Drosophila*, locomotor activity can be measured by different tools. Negative geotaxis performance is an innate escape behaviour in which adult flies ascend food vials as a response to tapping the vials. It could be used as a tool to evaluate the locomotor activity of *Drosophila*. This behaviour requires nervous system signaling commands via different biogenic amines.

Octopamine (OA) is one of these biogenic amines that acts as a neurohormone, neurotransmitter and neuromodulator in invertebrates. This catecholamine was first discovered in the salivary glands of *Octopus vulgaris*^3^. OA is synthesised from the amino acid tyrosine. Tyrosine is converted by decarboxylation to tyramine by the enzyme tyrosine decarboxylase, and then tyramine is converted by hydroxylation to OA with the help of tyramine beta-hydroxylase. OA orchestrates many diverse physiological and behavioural processes including aggression, fight-flight response, circadian rhythm and locomotion^4^. OA is essential for modulating the function of skeletal and visceral muscles^5^. OA in invertebrates has the same function of noradrenaline in vertebrates. OA exerts these functions by binding to and activating OA receptors belonging to the G protein-coupled receptor superfamily. Drosophila possesses four OA receptors: Oct*ß*1R (also referred as *oa2*: CG6919 ), Oct*ß*2R (CG33976, CG6989) and Oct*ß*3R (CG42244, CG7078) and Oct*α*R (also referred as *oamb,* CG3856)^6^. OctαR has two isoforms differ in the 3rd cytoplasmic loop and downstream sequence due to splicing of the last exon, both isoforms are highly enriched in mushroom bodies^7^. OA stimulates glycogenolysis, modifies muscle contraction, supports long-term flight and regulates “arousal” in the central nervous system^8^. Despite the potential relevance of OA for controlling locomotion, our knowledge about the role of each octopaminergic receptor still limited.

The GAL4/UAS system enables scientists to study either gain-of-function or loss-of-function of the gene (s) of interest^9^, facilitating studying gene expression in *Drosophila*^10^. The GAL4/UAS system has been used in RNA interference (RNAi) technology. This technology enables the knocking down a specific gene of interest.

The main goal of this work was to reveal which one of the *Drosophila* OA receptors is important for adult locomotion. We achieved this by knocking down each OA receptor everywhere within the insect body using a universal driver and performing the negative geotaxis assay with the corresponding adults. In order to explore the underlying mechanisms by which OA signalling controls locomotion, we asked whether the role of OA originates from its effect on the nervous system, more specifically from motor neurons or mechanosensory neurons sensing the gravity or muscular system. To address this point, specific GAL4 drivers were crossed to OA receptor (s) followed by evaluation of the climbing ability performance of F1 male adults. Finally, we examined the morphometric and skeletal muscle ultrastructure changes in the legs of adult flies that displayed the knocking down of OA receptors. The findings of the current study provide evidence that OA via Oct*α*R orchestrates nervous system commands with muscular system response, consequently controlling balanced locomotion and that the underlying mechanism by which OA controlling locomotion is a multifactorial complex process more than anticipated. Moreover, the current study is the first that link the leg muscles development with neurohormonal control. Information regarding biogenic amine receptors is very important for comparative, evolutionary physiology and biochemistry as well as serving as possible specific targets for insecticides.

## Results

**Figure 1:**
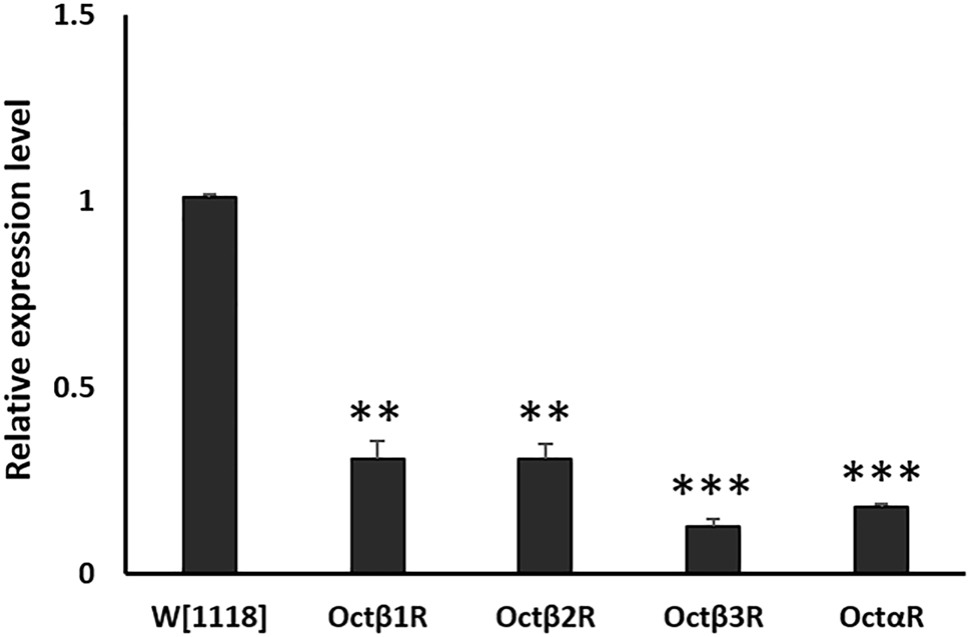
Use of qPCR to validate knock-down of octopaminergic receptors genes. Relative level of expression of octβ1R, octβ2R, octβ3R and octαR in knocked down vs control group. Mean ± SD; N = 3; **P ˂0.01, ***P ˂0.001.

**Figure 2:**
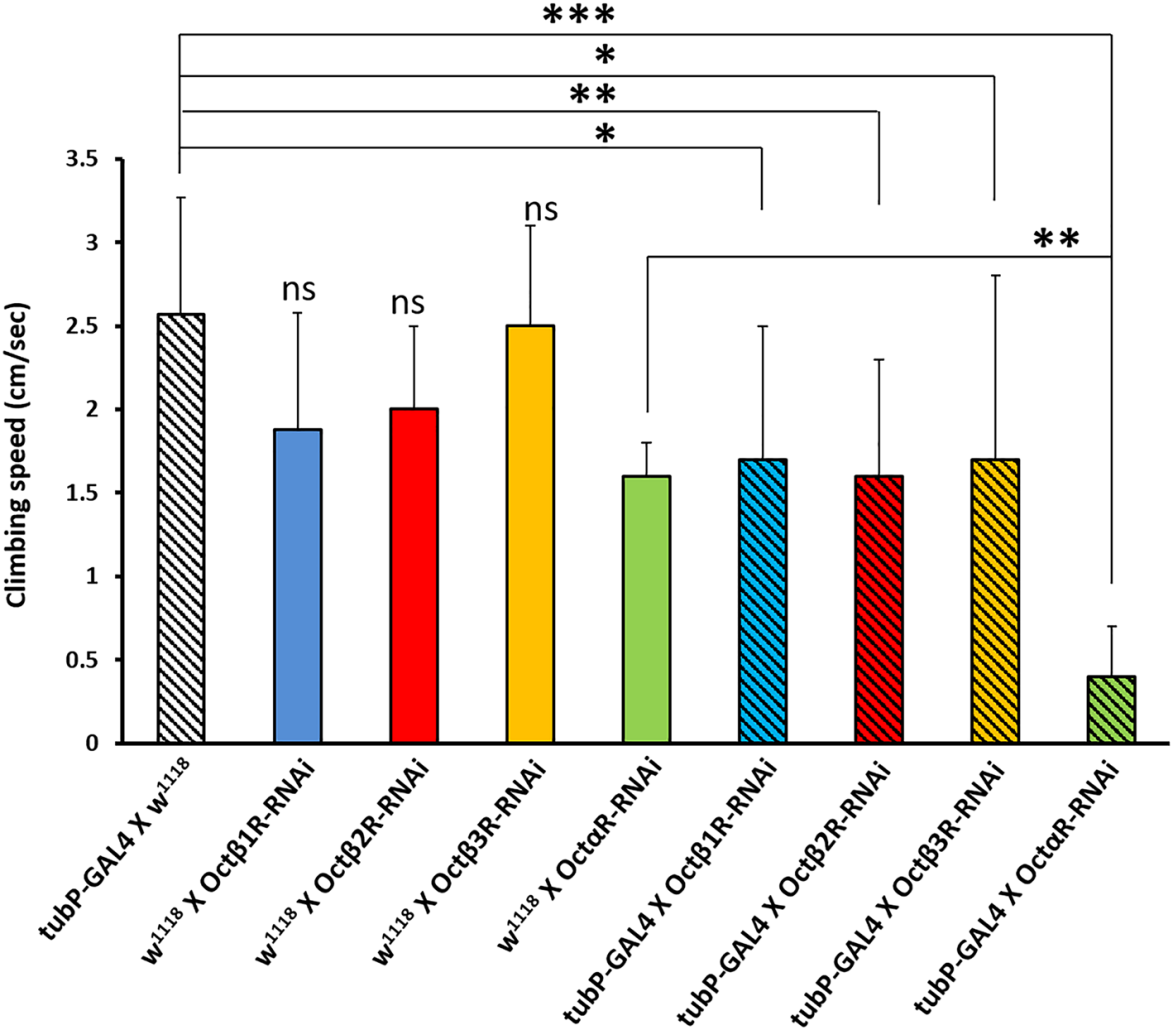
Climbing speed of adult *Drosophila* males that lost the expression of four different receptors receptive for octopamine using ubiquitous driver line *tubP-*GAL4 as compared to controls. The experiment was performed in triplicate. Mean ± SD; N = 10; *P ˂0.05, **P ˂0.01, ***P ˂0.001.

**Figure 3:**
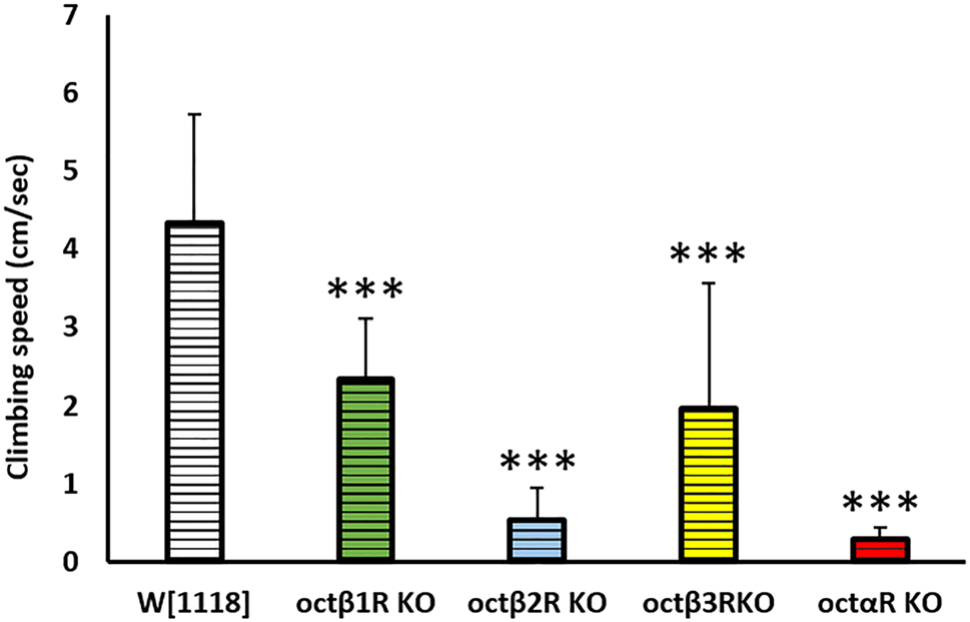
Climbing speed of male flies depleted of OA receptors. Three asterisks indicate high significance (Student t-test: *P* ˂ 0.001).

**Figure 4:**
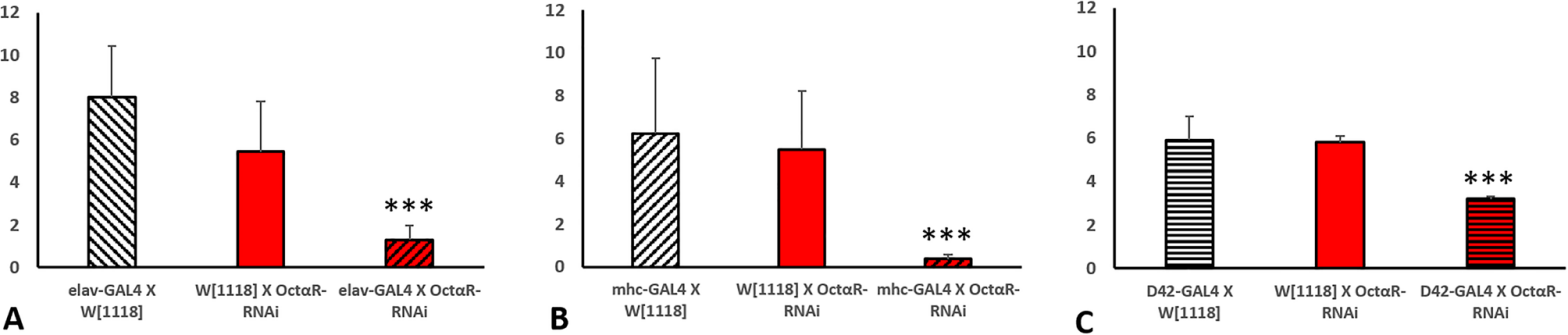
The climbing speed of *OctαR*-deficient adult *Drosophila* males (N = 10) as compared to controls. The experiment was performed in triplicate. RNAi-mediated gene kockdown was directed to the nervous system using *elav-GAL4* driver (**A**), muscular system using *mhc-GAL4* driver line (**B**) and to motor neurons using D42-GAL4 driver (**C**). Three asterisks indicate high significance (Student t-test: *P* ˂ 0.001).

In order to establish an overview of negative geotaxis performance relevant to OA receptors deficiency, RNAi lines targeted against the OA receptors were crossed with strong ubiquitous driver line *tubP-*GAL4 which was reported before to cause high percentage depletion in the expression level of target gene^11^ in most organs of the fly. In this study, we used RNAi construct (#31171) targeting both oct*α*R isoforms. The level of RNAi-based knock down of OA receptors in F1 adults was validated using qRT PCR (Fig. 1). Results showed significantly strong KD with low leaky expression (Student t-test: t-value was 8.82, 9.03, 11.28 and 10.74 for oct*β*1R, oct*β*2R, oct*β*3R and oct*α*R, respectively and P value was 0.001 for oct*β*1R, oct*β*2R and < 0.001 for oct*β*3R and oct*α*R). Negative geotaxis assay results obtained revealed that despite all receptors receptive for OA have significant adverse effect on climbing performance, Oct*α*R*-*deficient flies showed the most severe impairment, which was highly significantly different (*P* = 0.005) from that of control flies (Fig. 2). The control male flies showed the native characteristic strait-line walking behaviour to climb the cylinder. By contrast, Oct*α*R-deficient male flies showed impairment in moving up against gravity. Some flies attempted several times to climb up the cylinder for a short distance, but they failed to maintain their equilibrium and fell into the bottom of the cylinder. Furthermore, few flies showed complete defective locomotion behaviour. They remained on the cylinder bottom walking very slow throughout the experiment. The same climbing impairment was observed using KO lines (Fig. 3). Therefore, we focused on whether the underlying mechanism of octopamine signalling via Oct*α*R in climbing performance originated from the nervous system or muscular system. To achieve this, the Oct*α*R RNAi line was crossed to two driver lines *elav-*GAL4 and *mhc-*GAL4 lines, respectively. The results showed that targeting the RNAi effect of Oct*α*R either to the nervous system (*df* = 28, *t* = 10.396, *P *˂ 0.001) or muscular system significantly (*df* = 28, *t* = 6.401, *P *˂ 0.001) decreased the climbing ability of adult males compared with control flies (Fig. 4A,B). More specifically, when Oct*α*R knocked down exclusively in motor neurons-specific manner using GAL4 driver, *D42*-GAL4, the same impaired climbing behavior was observed (Fig. 4C).

**Figure 5:**
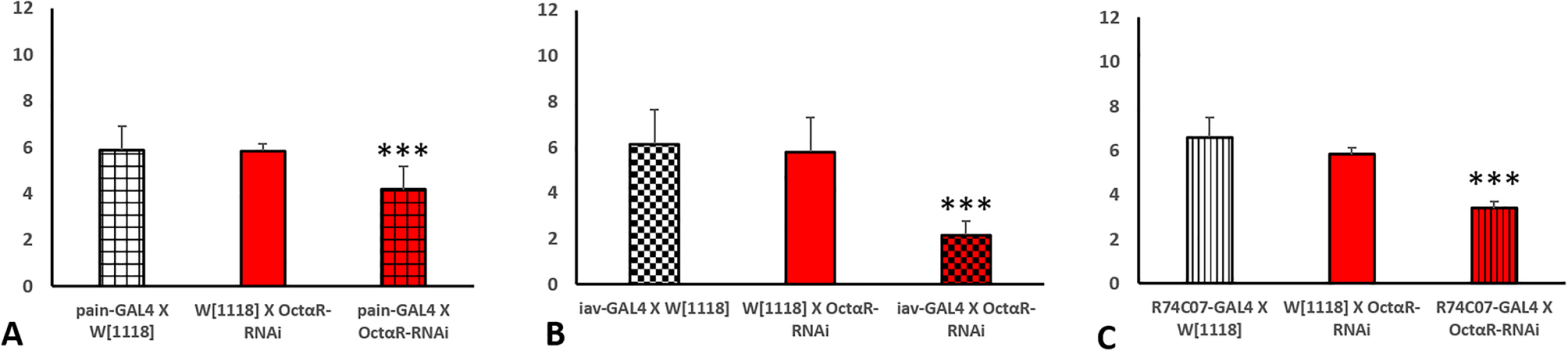
Negative geotaxis assay of adult male flies (N = 10) that lost the expression of octαR using specific mechanoreception GAL4 driver lines**.** Asterisks indicate significant differences among treatments (Student t-test).

As sensing the gravity through feedback of mechanoreceptors is also essential for balanced climbing behavior, we asked whether OctαR involved in this signal. To address this question, we knocked down octαR using two mechanoreceptor GAL4 drivers, *pain*-GAL4 and *iav*-GAL4. In addition, we used another driver line *R74C07*-GAL4 labelling mechanosensory neurons in parts of the body (eyes and posterior abdomen) other than antennae^12^. Results showed significant reduction in climbing performance in response to manipulation of Oct*α*R in mechano-sensing manner (Fig. 5).

**Figure 6:**
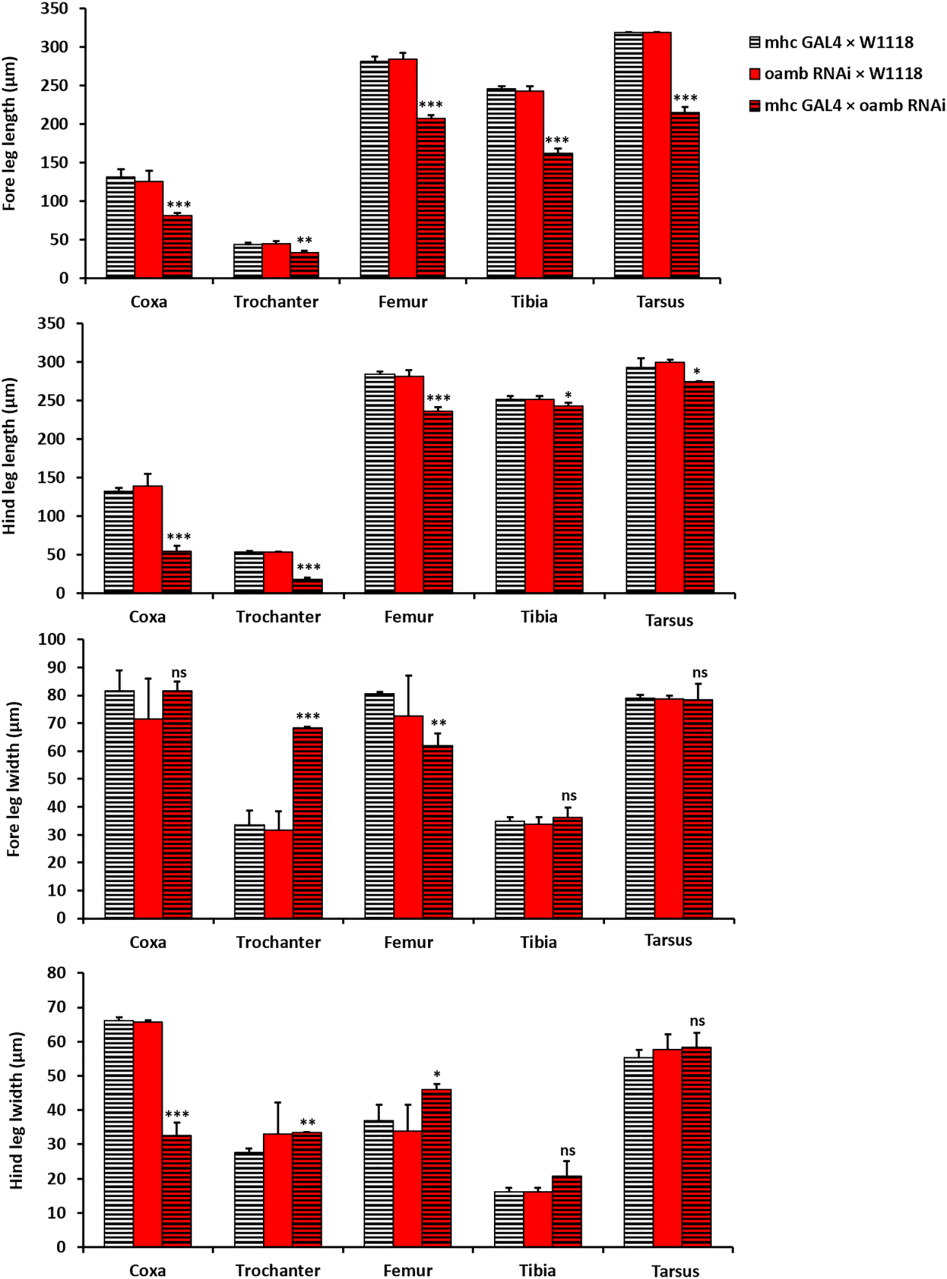
Length and Width of fore and hind legs' parts of adult flies that lost the expression of octαR in muscular system as well as matching control adult *Drosophila* males. Mean ± SD; N = 10; *P ˂0.05, **P ˂0.01, ***P ˂0.001.

**Figure 7:**
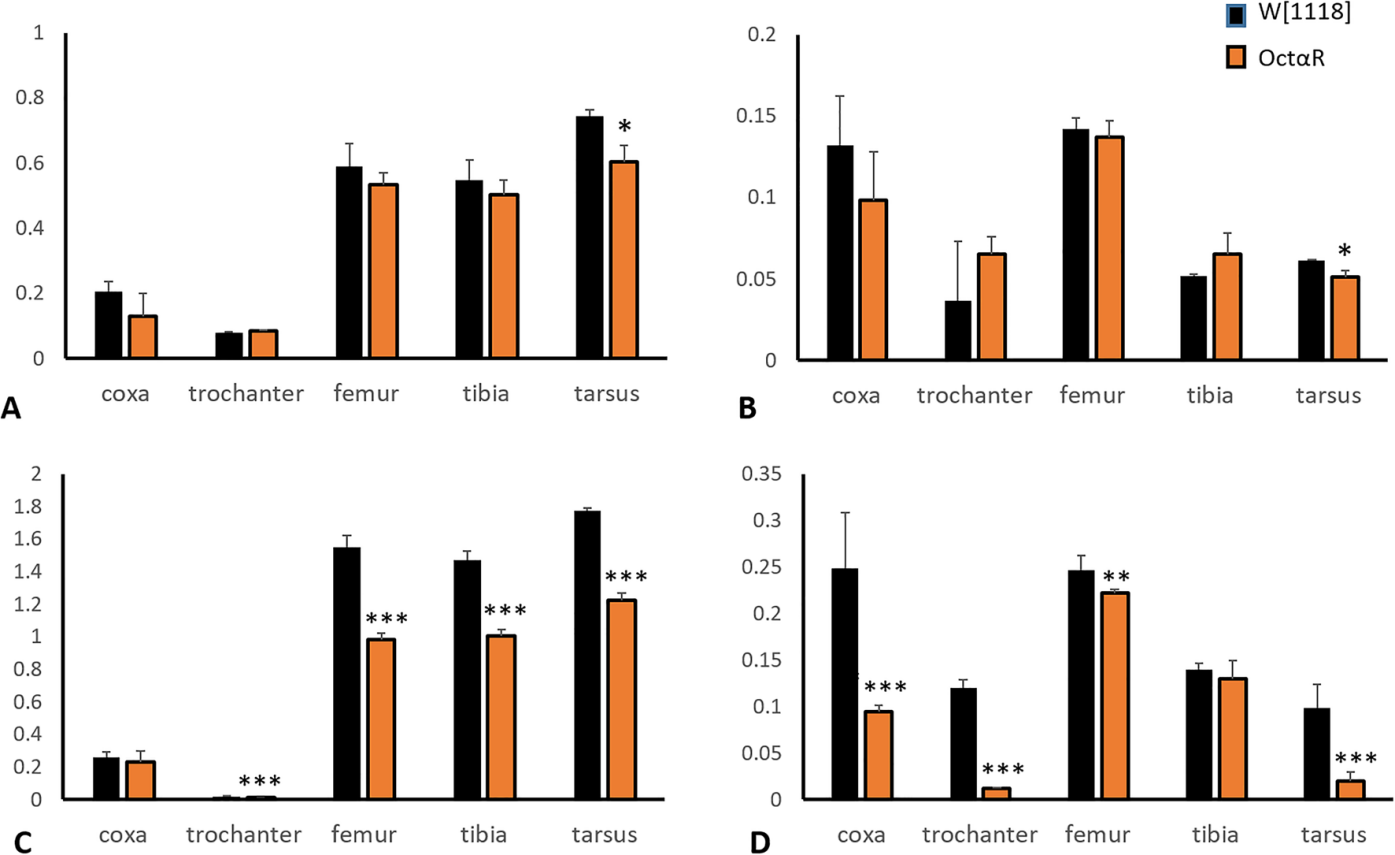
The effect of *OctαR* knock out on the length and width of fore and hind legs' parts as compared to those of w^1118^. Mean ± SD; N = 10; *P ˂0.05, **P ˂0.01, ***P ˂0.001.

To investigate whether knocking down Oct*α*R specifically in the muscular system cause morphometric changes in adult leg parts, the length and width of fore and hind leg segments were measured (Fig. 6). The results indicated that the lengths of all parts of forelegs (*t* = 8.013, *P* = 0.001 and *t* = 5.433, *P* = 0.006 for coxa and trochanter, respectively; *t* = 15.427, *P* = 0.000 for femur; *t* = 18.933, *P* = 0.000 for tibia and *t* = 22.232, *P* = 0.000 for tarsi) and hind legs (*t* = 16.324, *P* = 0.000 for coxa, *t* = 22.167, *P* = 0.000, for trochanter; *t* = 13.050, *P* = 0.000 for femur; *t* = 2.848, *P* = 0.046 for tibia and *t* = 2.842, *P* = 0.047 for tarsi) of *OctαR*-deficient flies were significantly different from those of control flies. In addition, the width of the trochanter and femur of the forelegs (*t* = 11.954, *P* = 0.000 for trochanter and *t* = 7.139, *P* = 0.002 for femur). In addition, the width of coxa (*t* = 14,613, *P* = 0.000), trochanter (*t* = 7.669, *P* = 0.002) and femur (*t* = 3.238, *P* = 0.032) of the hind legs were significantly different from those of control flies. In addition, Oct*α*R knock out mutant flies which showed significant reduction in fore and hind leg segments as compared to those of w^1118^ flies (Fig. 7).

**Figure 8:**
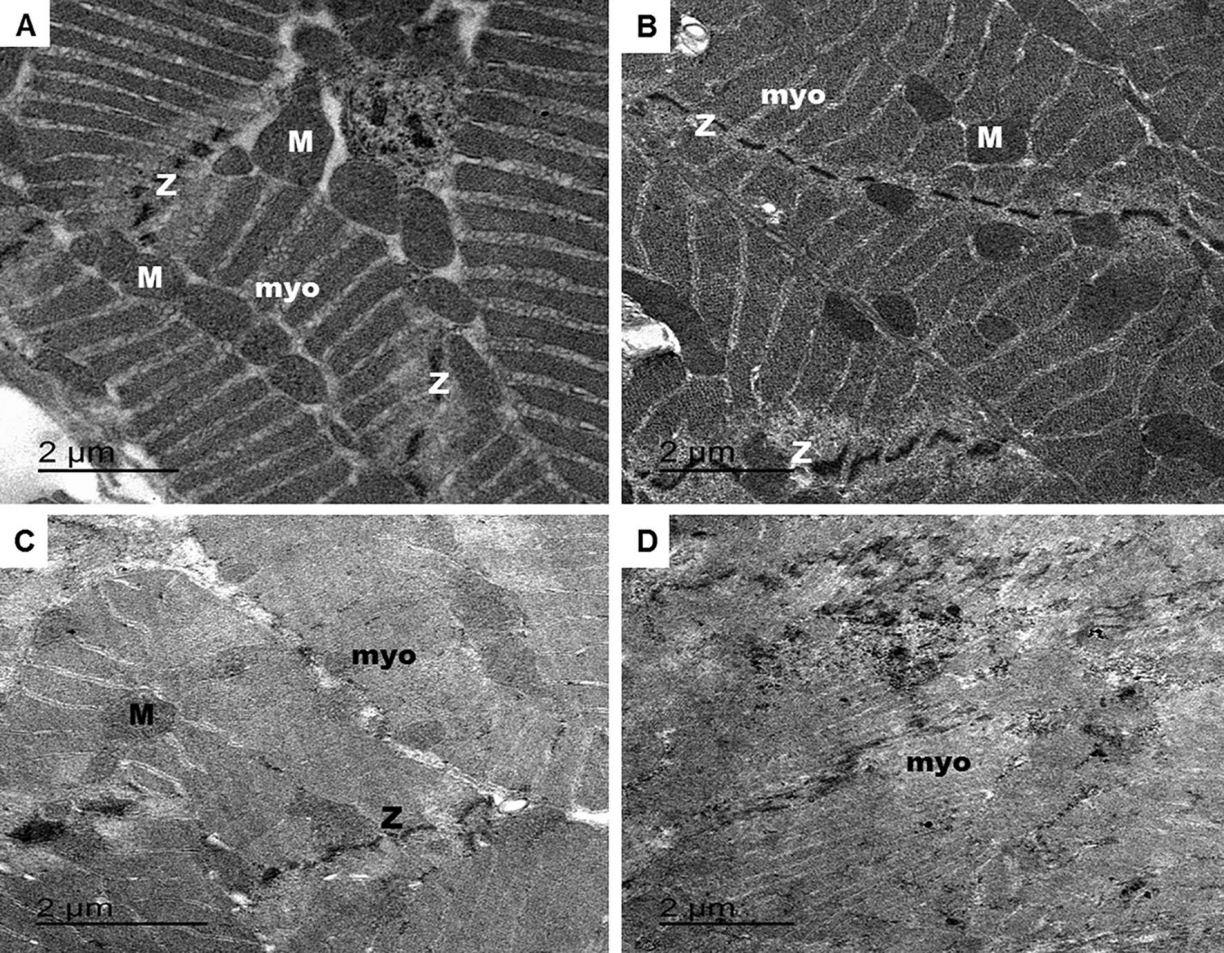
The effect of *OctαR* knocking down in the muscular system on the ultrastructure of the muscular tissue of the femur region of the hind legs as well as matching controls using mascular system driver, mhc-GAL4. (**A**,**B**) TEM micrograph of male flies of the controls of RNAi experiment. The muscles display features typical of insect skeletal muscle. (**C**,**D**) TEM micrograph of *OctαR* deficient flies leg muscles. *M* mitochondria, *Myo* myofibrils, *Z* z-line.

In this study, ultrastructure examination of femur region of the metathoracic leg of control flies revealed that the tubular oblique muscles consist of spindle-shaped myofibrils, arranged in parallel and had the known striated pattern of successive sarcomeres (Fig. 8A,B). All muscle fibres were ensheathed in a sarcolemma. The sarcolemma invaginates into fibres at regular distances. Each sarcomere extends between adjacent Z-discs of the myofibril. The Z line generally looks like a series of dense bodies and disconnected through the sarcomere. Their roundish mitochondria are present between the myofilaments and contain an electron-dense matrix. When Oct*α*R knocked down in the muscular system using *mhc*-GAL4, the muscles displayed disorganisation of all components. The sarcomeres and the entire myofibrils showed loss of striations and structure (Fig. 8C,D). The sarcoplasmic reticulum around each myofibril was noticeably disintegrated such that division of the muscle fibres into myofibrils was indistinct. The myofibrils broke down such that there were spaces in the muscle fibre. The Z line split into smaller fragments that were dispersed among the sarcomeres. Although mitochondria were distributed along damaged myofibrils, some were enlarged with an irregular contour, some were small with a rounded contour and others were absent from certain areas of the muscle fibre. Their cristae were electron lucent.

## Discussion

OA is a catecholamine that acts as a neuromodulator in invertebrates^4,13–15^. The role of neuromodulators was explained by “orchestration hypothesis,” which assumed that neuromodulator release into specific neuropils configures neural commands to produce a coordinated network activity^16^. OA mediates its function by binding to G protein-coupled receptors, which included cyclic AMP production or Ca^2+^ release. OA regulates many physiological processes including aggression, fight-flight response, circadian retime and locomotion^17–20^.

Each leg segment of the multi-jointed legs of adult insects contains a stereotyped arrangement of muscles. Contractions of these muscles through motor neurons control the coordination of locomotion. OA plays an important role in this process. In locusts, OA is delivered in time for enhancement of leg muscle contraction^21^. In this study, we focused on the role played by octopaminergic receptors in balanced locomotion using RNAi of each receptor with different GAL4 drivers. Targeted RNAi-mediated knockdown using the tubulin-promoter GAL4 driver showed that *OctαR*-deficient flies had the weakest performance in the negative geotaxis assay. This result was confirmed by evaluating climbing performance in OA receptors knocked out (KO) mutant lines which was lower than wild type w^1118^. When Oct*α*R deficiency was directed to the muscular, nervous system and more specifically motor neurons, we observed significant impairment in negative geotaxis performance. The walking speed of Oct*α*R*-*deficient flies was lower than that of control flies*.* Moreover, most of leg parts of *OctαR-*deficient flies was smaller than those of control flies. Smaller leg parts was also observed in Oct*α*R-kocked out adults as compared to w^1118^.

Sense gravity via mechanosensory structure located in the antenna is important for spatial orientation for gravity negative geotaxis^22^. Many genes are involved in this process such as painless (*pain*)^23^ and inactive (*iav*)^24,25^ which are expressed in Johnston’s organ chordotonal neurons and femoral chordotonal organs and is required for normal hearing as well as gravity sensing^22^. We asked whether octαR play role in orchestration of feedback signals received via mechanosensory receptors required for normal climbing behavior response. To address this point, we knocked down octαR in two gravity-sensing manner using mechanoreceptor GAL4 driver line namely *pain*-GAL4 and *iav*-GAL4. In addition, we used another driver line *R74C07*-GAL4 labelling mechanosensory neurons in parts of the body (eyes and posterior abdomen) other than antennae^12^. Results showed reduction in climbing performance in response to manipulation of Oct*α*R in mechano-sensing manner which may provide more additional underlying mechanism of OA signalling control of climbing behaviour via Oct*α*R. It was reported before that OA increased responses of mechanically stimuli^26–28^. Torkkeli et al.^29^ stated that the OA receptor expressed in mushroom bodies increases the sensitivity in the spider *Cupiennius salei* mechanosensory neurons. These findings are interesting as they provide evidence that *OctαR* acts in leg muscles as a neuromodulator/neurotransmitter that is important for the development of normal leg muscle architecture and size and for the coordination of mechanical stimuli sensation with muscle response which is required for balanced locomotion. A possible explanation for these changes is based on the known physiological role of Oct*α*R mediated signalling that induces Ca^2+^ oscillations due to Ca^2+^ release from intracellular stores^30^. This reaction is controlled by kinase and phosphatase activities^31^. Furthermore, blocking phosphatase activity in Oct*α*R expressing cells completely abolished Ca^2+^ oscillations (LIT). In mammals, it has been shown that Ca^2+^-dependent pathways control muscle development^32^. Lowering intracellular calcium levels inhibits the differentiation of skeletal myoblasts into mature myotubes^33^. Moreover, it has been shown in mammals that adrenergic receptors signalling regulated myoblast differentiation^34,35^. This might explain, why the lack of Oct*α*R-mediated signalling events during development leads to the observed structural changes. The neuromodulatory role of OA in skeletal and visceral muscle contraction was reported before^36^. Oct*α*R expression was reported in adult *Drosophila* leg muscles^37^. *OctαR*, the invertebrate counterpart of mammalian α-adrenergic receptor, is also expressed in the oviduct muscles of female insects and regulates their contraction by elevating the cytosolic Ca^+2^ level^38^ and in the tracheal system^37^. OA through Oct*α*R regulates other physiological processes such the induction of insulin release from insulin-producing cells causing changes in the amount of daily sleep and changes in fat storage, leading to lean adult flies^39,40^.

To verify the role of Oct*α*R in the formation of normal leg muscle architecture, leg muscles of male *Drosophila* were analysed using transmission electron microscopy. The results revealed that in *OctαR*-deficient flies, the leg muscles displayed abnormal morphologies of sarcomeres, disorganised myofibrils and mitochondrial abnormalities. For these reasons, male *Drosophila* flies hypothesize to show severe defective locomotion behaviour. The sarcomeres and the entire myofibrils showed loss of striations and structure. The sarcoplasmic reticulum around each myofibril was noticeably disintegrated; consequently, division of the muscle fibres into myofibrils was indistinct. Moreover, the myofibrils broke down forming spaces in the muscle fibre. The thickness of myofibrils that could be observed in the leg muscles of Oct*α*R-deficient flies was significantly less than that the leg muscles of control flies. Z-lines as anchoring structures for myofilaments dictate the final length of sarcomeres. Z-lines would be expected to participate in the organisation of myofilaments during the initial stages of myofibril assembly. The results of this study showed that Z-lines within the leg muscles of Oct*α*R-deficient flies were split into smaller fragments that were dispersed among the sarcomeres, causing loss of the normal muscle architecture and consequently impairment of the negative geotaxis performance of corresponding flies. Recently, Sujkowski et al.^44^ reported that Oct*α*R expression in *Drosophila* is required for adaptations in skeletal muscles in legs.

Mitochondria play an important role in the muscular system. In *Drosophila*, myofibers have a greater dependency on mitochondria and lipid oxidation and, thus, the numbers of healthy mitochondria can influence the capacity to maintain muscle mass and function. In skeletal muscles, mitochondria regulate energy haemostasis by producing ATPs required for muscle contraction through oxidative phosphorylation. In addition, mitochondria contribute to Ca^+2^ homeostasis^45,46^ redox signalling^47,48^, release of pro-apoptotic factors^49^, synthesis of haeme molecule and regulation of nuclear gene expression^50,51^. In this study, ultrastructural changes were detected in the shape of mitochondria within the leg muscles of *OctαR*-deficient flies. The mitochondria were electron lucent, enlarged with an irregular contour or small with a rounded contour and others were absent from certain areas of the muscle fibre. These changes could be the reason behind the observed disorder of muscles. Ultrastructure changes in mitochondria occur in muscle disorders^52–55^, particularly in muscular dystrophy, while in degenerating fibres, the number of mitochondria is reduced and they disappear from severely atrophied muscle fibres.

Taken together, the data presented in this study reveal that octopamine signalling via Oct*α*R plays an essential role in adult *Drosophila* movement ability as well as in normal leg muscle architecture formation and interorgan communication between the nervous system commands to motor neurons and muscular tissue responses. It became apparent that information regarding biogenic amine receptors is very important form many points of view including comparative, evolutionary physiology and biochemistry as well as serving as possible specific targets for insecticides.

## Contribution to the field

Biogenic amines act as neurohormones, neuromodulators and neurotransmitters controlling many physiological processes in both vertebrates and invertebrates. Some commercially available pesticides act on biogenic amines such as Rotenone, neonicotinoids and formamidine. Biogenic amines-based pesticides have adverse effects on human, especially nervous system causing neurological diseases. For this reason, the biogenic amine octopamine and its related receptors represent good insecticide targets as being exclusive to invertebrates. Consequently, detailed information about the contribution of single receptor in vital physiological and behavioral processes may help to choose the most effective target for specific insecticides design tools. In this study, we focus on the contribution of octopaminergic receptors in locomotion, as a crucial behavior for survival. Results of tissue specific RNAi-based experiments revealed that *OctαR*-deficient flies showed impaired negative geotaxis and leg muscles atrophy. In addition, this study revealed for the first time, the role of *OctαR* in the development of normal leg muscles architecture. Previous studies stated that agonists and antagonists for octopamine receptors act as potential targets for insecticides^56^, here our results specify OA receptor OctαR as a possible target for molecular docking for synthesis of specific insecticides due to the essential role of this receptor in locomotion and skeletal muscle development. In addition to this, the data provide new insights into what may be a potential future prospect for human muscle atrophy therapeutics by targeting the α-adrenergic receptors, the counterparts of invertebrate *OctαR*.

## Materials and methods

### *Drosophila* strains and rearing

All *Drosophila* lines (Table 1) were obtained from Bloomington Drosophila Stock Center (Bloomington Stock Centre, Indiana University, Bloomington, USA). These lines included RNAi effector lines generated by the Transgenic RNAi Project for the knockdown of *Drosophila* OA receptors (RNAi for DmOct*α*R#31171, DmOct*β*1R# 31106, DmOct*β*2R# 34673 and DmOct*β*3R# 31108) and GAL4 driver lines (*tubP-*GAL4 #5138, *elav-*GAL4 # 8765, *Mhc-*GAL4#55133, *pain* GAL4 # 27894, *D42*-GAL4 #8816, *iav*-GAL4 #52273 and R74C07-GAL4 (*meru*-GAL4) # 39847). First, the efficiency of knocking down of RNAi transgenes was validated using qRT PCR as described before^57^. Primer sequences are as follow: DmOct*α*R forward 5ʹ-CGG TTA ACG CCA GCA AGT G-3 ʹ; DmOct*α*R reverse 5ʹ-AAG CTG CAC GAA ATA GCT GC-3ʹ, DmOct*β*1R forward 5ʹ-GGC AAC GAG TAA CGG TTT GG-3ʹ; DmOct*β*1R reverse 5ʹ-TCA TGG TAA TGG TCA CGG GC-3ʹ, DmOct*β*2R forward 5ʹ-TCC TGT GGT ACA CAC TCT CCA-3 ʹ; DmOct*β*2R reverse 5ʹ-CCA CCA ATT GCA GAA CAG GC-3ʹ, DmOct*β*3R forward 5ʹ-TGT GGT CAA CAA GGC CTA CG-3ʹ; DmOct*β*3R reverse 5ʹ-GTG TTC GGC GCT GTT AAG GA-3ʹ, and a house keeping gene rpl32 forward 5ʹ-CCG CTT CAA GGG ACA GTA TC-3ʹ; rpl32 reverse 5ʹ-GAC AAT CTC CTT GCG CTT CT-3ʹ. Then, for knocking down octopamine receptors, virgin females carrying GAL4 driver was crossed with: (i) UAS-RNAi effector lines males (ii) W^1118^ (#3605) males to produce offspring as the first control for RNAi experiment. Additionally, W^1118^ virgin females were crossed with males carrying RNAi transgenes to serve as the second control for RNAi experiment. At least two available constructs for the same receptor-coding gene have been tested to be sure the observed phenotype is not due to off-target effects and the results presented in the manuscript were to the strongest construct which show high significant difference. Drosophila mutants for octopamine receptors (DmOctαR KO#83030, DmOctβ1R KO# 83035, DmOctβ2R KO# 82785 and DmOctβ3R KO# 83046) were obtained from Bloomington Drosophila Stock Center. All lines were reared on standard *Drosophila* medium (14–15 g agar, 18.5 g yeast, 61 g glucose, 30.5 g sucrose, 101 g corn meal/L) at 25 °C except for F1 of the crossings was kept at 27–29 °C to enhance the RNA interference effect. All lines were kept at 50%-60% relative humidity with an 18/6-h light/dark cycle.

**Table 1:** Stock number, name, genotype and usage of *Drosophila* lines used in this study.

Stock#	Name	Genotype	Function
3605	Wild type	w[1118]	
5138	*tubP*-GAL4	y[1] w[*]; P{w[+ mC] = tubP-GAL4}LL7/TM3, Sb[1] Ser[1]	ubiquitously GAL4 driver
8765	*elav*-GAL4	P{w[+ mC] = GAL4-elav.L}2/CyO	Nervous system GAL4 driver
8816	*D42*-GAL4	w[*]; P{w[+ mW.hs] = GawB}D42	Motor neurons GAL4 driver
27894	*Pain*-GAL4	w[*]; P{w[+ mW.hs] = GawB}pain[GAL4]	Multidendritic neurons, chordotonal neurons, a subset of cells in the CNS and a subset of sensory neurons in the antennal-maxillary complex GAL4 driver
39847	*meru*-GAL4	w[1118]; P{y[+ t7.7] w[+ mC] = GMR74C07-GAL4}attP2	Mechanosensory GAL4 driver
52273	*Iav*-GAL4	P{iav-GAL4.K}3	Chordotonal neurons GAL4 driver
55133	*mhc*-GAL4	w[*]; P{w[+ mC] = Mhc-GAL4.K}2/TM3, Sb[1]	Muscular system GAL4 driver
31106	RNAi-UAS-*Octβ1R*	y[1] v[1]; P{y[+ t7.7] v[+ t1.8] = TRiP.JF01571}attP2	Expresses dsRNA for RNAi of Octbeta1R under UAS control
31108	RNAi-UAS-*Octβ3R*	y[1] v[1]; P{y[+ t7.7] v[+ t1.8] = TRiP.JF01573}attP2/TM3, Sb[1]	Expresses dsRNA for RNAi of Octbeta3R under UAS control
31171	RNAi-UAS-*OctαR*	y[1] v[1]; P{y[+ t7.7] v[+ t1.8] = TRiP.JF01673}attP2	Expresses dsRNA for RNAi of OctalphaR under UAS control
34673	RNAi-UAS-*Octβ2R*	y[1] sc[*] v[1] sev[21]; P{y[+ t7.7] v[+ t1.8] = TRiP.HMS01151}attP2	Expresses dsRNA for RNAi of Octbeta2R under UAS control
82785	*Octβ2R* KO	y[1]; M{v[+ t1.8] = WKO.1-H1}ZH-86Fb	*Octβ2R* knocked-out mutant line
83030	*OctαR* KO	y[1]; M{v[+ t1.8] = WKO.1-F1}ZH-86Fb	*OctαR* knocked-out mutant line
83035	Octβ1R KO	y[1]; M{v[+ t1.8] = WKO.1-G1}ZH-86Fb	*Octβ1R* knocked-out mutant line
83046	Octβ3R KO	y[1] v[1]; M{v[+ t1.8] = WKO.1-A2}ZH-86Fb	*Octβ3R* knocked-out mutant line

### Climbing assay (negative geotaxis)

A negative geotaxis assay was performed using only male adult flies to avoid the effect of weight changes in adult female flies due to the physiological process including ovulation and oogenesis. Briefly, 10 males were placed into a 100 mL empty glass cylinder. The flies were tapped to the bottom of the cylinder and left to climb the cylinder. This procedure was repeated five times. The upward movement of the flies was recorded until the last climbing fly reached the top of the vial. The upward movement of flies was recorded with a digital video camera. The videos were cut into snapshots at the rate of 5 frames/second. These frames were analysed using ImageJ 1.46r software (National Institutes of Health, Bethesda, MD, USA) to trace the upward paths of the flies. The walking speed was calculated by dividing the length of the path (cm) by the time required in each path (s)^58,59^.

### Effect of OA receptor knockdown on climbing ability

OA receptors were silenced using the GAL4/UAS system. First, the universal driver tubulin promoter GAL4 (*tubP-*GAL4), which induces gene repression in almost all insect’s body cells, was independently crossed to the UAS-*OctaR*, *Octß1R*,* Octß2R* or *Octß3R* RNAi effector lines to detect the OA receptor which is strongly involved in climbing performance. The climbing ability of ten F1 adult males was studied as described above. Adult F1 males of crossing between *w*^*1118*^ and : (i) *tubP-*GAL4 and (ii) RNAi lines were used as controls. Then to explore the root cause of the behaviour performance impairment, we down regulate Oct*a*R using specific tissue driver lines: nervous system-specific driver *elav-*GAL4, motorneurons driver D42-GAL4, muscular system-specific driver myosin heavy chain (*Mhc-*GAL4), gravity-sensing drivers *Pain*-GAL4, *iav*-GAL4^22^ and *meru*-GAL4. GAL4 driver line were crossed to UAS-Oct*a*R RNAi effector line and the climbing performance of F1 males (N = 10) was evaluated as mentioned above. In addition, the effect of OA receptors ablation on climbing performance was investigated using *Drosophila* knocked out (KO) lines for OA receptors.

### Morphometric analysis of adult legs

Fore and hind legs were removed by forceps from F1 of UAS-*OctαR*-dsRNAi × *mhc*-GAL4. Then transferred to glass slides using a fine brush for microscopic examination using an Olympus BX61 light microscope at magnification 2.5 × 10. Photographs were taken and the length and width of each leg segment (coxa, trochanter, femur, tibia and tarsi) were measured using ImageJ software. In parallel, F1 of w^1118^ crossed to: (i) OctαR-dsRNAi and (ii) GAL4-mhc was used as controls for RNAi experiment. Additionally, morphometric analysis of Oct*α*R KO adult legs was done. W^1118^ was used as control for knock out (KO) experiment.

### Transmission electron microscopy

Ten hind legs from UAS-Oct*α*R-RNAi × *mhc*-GAL4 adult offspring were removed by forceps. Then, the legs were fixed in 5% glutaraldehyde in sodium cacodylate buffer (pH 7.4) for 12–24 h. The specimens were washed and fixed overnight in 15% buffered osmium tetroxide. Samples were incubated overnight in 0.5% aqueous uranyl acetate solution, then dehydrated, filtered and embedded in spur resin^60^. Semi-thin (1 μm) sections were stained with methylene blue Atur II and examined using an Olympus BX61 light microscope. Ultra-thin sections (60 nm) were cut using an ultramicrotome (Ultracut S, Leica), and stained with 2.5% uranyl acetate and lead citrate and were examined using JEOL, JEM 100-SX electron microscope.

### Statistical analysis

Data represented as (Mean ± SD). Normality of the data was tested with the Kolmogorov-Smirnov test. Two sample t-test was used to compare between control and other groups. For all statistical tests p-value > 0.05 is considered not-significant. *p*-value < 0.05 is considered significant. p-value < 0.001 is considered highly significant. All data were analyzed using, Minitab software package version 19.0 and Microsoft Excel 365.

## Data Availability

The datasets generated during and/or analysed during the current study are available from the corresponding author on reasonable request.
